# The Interplay between Radioresistant Caco-2 Cells and the Immune System Increases Epithelial Layer Permeability and Alters Signaling Protein Spectrum

**DOI:** 10.3389/fimmu.2017.00223

**Published:** 2017-03-03

**Authors:** Jacopo Morini, Gabriele Babini, Sofia Barbieri, Giorgio Baiocco, Andrea Ottolenghi

**Affiliations:** ^1^Laboratory of Radiobiology and Radiation Biophysics, Department of Physics, University of Pavia, Pavia, Italy

**Keywords:** ionizing radiation, cytokines, Caco-2 cells, peripheral blood mononuclear cells, trans-epithelial electrical resistance

## Abstract

Colorectal cancer is one of the most frequent type of cancer, with a higher incidence in the developed countries. Colorectal cancer is usually managed with both surgeries, chemotherapy and radiotherapy. Radiotherapy has the well-known advantage of targeting the tumor, minimizing normal tissue exposure. Nevertheless, during radiation treatment, exposure of healthy tissues is of great concern, in particular because of the effects on the intestinal barrier functions and on cells belonging to the immune system. The functional role of intestinal barrier in avoiding paracellular trafficking and controlling bacterial spread from gut it is well known and it is due to the presence of tight junction complexes. However, intestinal barrier is fundamental in participating to the interplay with immune system, especially considering the gut-associated lymphoid tissue. Until few years ago, radiotherapy was considered to bear only a depressive action on the immune system. However, it is now recognized that the release of pro-inflammatory signals and phenotypic changes in tumoral cells due to ionizing radiation could trigger the immune system against the tumor. In this work, we address how intestinal barrier functions are perturbed by X-ray doses in the range 0–10 Gy, focusing on the interplay between tumoral cells and the immune system. To this aim, we adopted a coculture model in which Caco-2 cells can be grown in presence/absence of peripheral blood mononuclear cells (PBMC). We focused our attention on changes in the proliferation, trans-epithelial electrical resistance (TEER), cytokine release, and proteins of the junctional complexes. Our results indicate a high radioresistance of Caco-2 in the investigated dose range, and an increased permeability of the tumoral cell layer due to the presence of PBMC. This is found to be correlated with activation of PBMC, inhibiting the apoptotic pathway, with the enhancement of cytokine release and with variation of tight junction scaffold protein expression levels, assumed to be related to IFN-γ- and TNF-α-mediated signaling.

## Introduction

Colorectal cancer is the third most frequent type of cancer (after lung and breast cancers), with an incidence of 1,360,602 cases (9.7%) in 2012 all over the world, with a higher incidence for males (10.1%) compared to females (9.2%) (Global Cancer Observatory, International Agency for Research on Cancer, WHO, http://gco.iarc.fr). The estimated number of deaths due to colorectal cancer is 693,933 (8.5%).

Management of colorectal cancer is routinely performed through either surgeries, chemotherapy or radiotherapy (1). The advantage of radiotherapy is the localized delivery of radiation which allows, in the majority of cases, to avoid the systemic adverse reactions typical of chemotherapy. Although the systemic complications are reduced during radiotherapy, consequences can arise in the surrounding normal tissues, i.e., activation of inflammatory pathways, direct damage to healthy cells and non-targeted effects (2–4).

Among the effects elicited by radiation dose delivered during radiotherapy for colorectal cancers, two main issues deserve deep investigation, namely the interplay between tumoral cells and the immune system, and the changes in the intestinal permeability.

One of the most important functions of the intestinal epithelium is to create an impermeable barrier, in order to avoid paracellular passage of molecules and solutes. Impermeability is maintained thanks to the action of junctional complexes between epithelial cells (i.e., tight junctions and adherens junction). These complexes join together with the ones present on the plasma membrane of the surrounding cells creating a barrier, and are furthermore responsible of the polarity of the cellular monolayer (5).

The fundamental proteins that contribute in the formation of tight junctions are occludin and claudin-1, and all the proteins acting as adaptors between the junction and the cytoskeleton (ZO family members, afadin, CD2AP). Besides their function in creating junctions, it is well known that all these proteins play a crucial role in the transduction of signals inside the cell; a lot of these proteins are in fact misregulated in different types of cancer and are frequently responsible of signals able to orchestrate both proliferation and metastasis formation (6–8). As an example, claudin-1 expression was found to be reduced in breast (9, 10) and in colon cancer; in colon cancer it was also associated with recurrence and poor prognosis (11).

There is an increasing evidence that intercellular junction functioning and stability (e.g., tight junction, adherens junction, etc.) can be altered by irradiation (12). It has been demonstrated that the tight junction structure becomes disorganized when exposed to radiation, and this effect leads to an increased permeability of the monolayer (12). Deirò de Carvalho and colleagues showed that ionizing radiation (IR) exposure causes redistribution of the principal junctional proteins, leading to disassembly and loss of function of junctional complexes in Caco-2 cells (5). Moreover, Moyes and colleagues confirmed radiation-induced permeability changes, accompanied by an increased microparticle uptake (13).

Over the last 30 years, Caco-2 cell lines have been widely used as a model of intestinal barrier. Caco-2 cells derive from human colon adenocarcinoma: although they are tumoral cells, Caco-2 show the ability to differentiate in culture to create a functional polarized monolayer (14). Such ability to create a functional monolayer allowed the study of membrane functions when cells are grown on a porous support. This type of cell culture allows to create a difference among the two sides of the monolayer, improving differentiation and creating a gold standard for physiological intestinal transport and toxicity studies (14).

Being the culturing of Caco-2 cells on porous membrane an optimum *in vitro* model of intestinal barrier, an upgrade of this model is the coculture of Caco-2 cells with other cell types; this setup has been used in several studies to measure the interplay between different cell types (15), therefore adopted to highlight how Caco-2 response to exogenous stimuli is modified by coculture with respect to Caco-2 cells alone. For example, the Caco-2 coculture setup is common in such studies aiming to identify the complex cross talk between with the immune system due to the presence of non-pathogenic bacteria (16).

In their study, Pozo-Rubio and colleagues assessed the level of different cytokines in a Caco-2/peripheral blood mononuclear cells (PBMC) coculture with bifidobacteria stimulating the top layer of Caco-2 cells. They demonstrated that the cytokines secretion profile was completely different when compared with the one obtained after stimulation in absence of PBMC (17).

Other studies aimed especially at understanding the different response of Caco-2 cells to non-pathogenic and pathogenic bacteria. It was observed by Haller et al. that Caco-2 cells show a discriminative activation depending on treatment with lipopolysaccharide from enteropathogenic *Escherichia coli* spp. or non-enteropathogenic bacteria, i.e., *E. coli* spp., *Lactobacillus johnsonii*, and *Lactobacillus sakei* (18).

The aim of our work was to study the effect of X-ray irradiation in Caco-2 cells alone or cocultured with PBMC. In particular, we focused our study on modification of monolayer permeability, cell proliferation, and cytokine release. Finally, we tried to extrapolate the influence of cytokine spectra alteration on Caco-2 cell permeability and related tight junction pathways.

## Materials and Methods

### Cell Culture and Coculture Setup

Caco-2 cells were cultured in T75 flasks, 75 cm^2^, in RPMI 1640 (Lonza) supplemented with 10% fetal bovine serum (Lonza), 2 mM l-glutamine (Lonza), 100 IU/ml penicillin, and 100 μg/ml streptomycin (Lonza).

Human PBMC were isolated from healthy volunteers after written informed consent, in accordance with the Declaration of Helsinki. PBMC were isolated from heparinized blood using Ficoll Histopaque-1077 (Sigma) gradient. After separation, PBMC were washed twice with RPMI 1640 (Lonza) then grown in T25 flasks, 25 cm^2^, in RPMI 1640 supplemented with 10% fetal bovine serum (Lonza), 2 mM l-glutamine (Lonza), 100 IU/ml penicillin, and 100 μg/ml streptomycin (Lonza).

Cells were routinely grown at 37°C in a humidified atmosphere containing 5% CO_2_.

Human PBMC were collected on the day of the experiment and 2 × 10^6 ^cells/ml were put in the bottom compartment of the coculture 30 min after the irradiation of Caco-2 cells.

### Irradiation Setup

Exposures of Caco-2 cells to X-rays were performed with a 6 MV LINAC (Varian) at the IRCCS S. Maugeri (Pavia, Italy). Cells were irradiated with a dose rate of 3 Gy/min and doses in the range 2–10 Gy at room temperature. Sham-irradiated cells experienced the same environmental/procedural conditions of the irradiated ones, without entering the irradiation room (0 Gy).

### Cell Viability Assays

Caco-2 cell growth was determined with the 3-(4,5-dimethylthiazol-2-yl)-2,5-diphenyltetrazolium bromide (MTT) assay as previously described (19) and with Trypan Blue assay.

For the determination of cell viability through MTT assay after radiation exposure, cells were seeded 24 h before irradiation in 24-wells plates (2 × 10^5^) in complete medium. Twenty-four and forty-eight hours after irradiation, 80 μl of 5 mg/ml MTT solution (Sigma) were added and kept in the incubator for 3 h then formazan crystals were dissolved with DMSO (Sigma). Results are always shown with respect to the corresponding sham condition, which is normalized to 100%.

For cell death determination through Trypan Blue assay, cells were irradiated as previously described. After 24 and 48 h, 50 μl of cell suspension were mixed with 50 μl of Trypan Blue dye (Amresco) and incubated for 3 min at room temperature. Unstained (viable) and stained (non-viable) cells were counted in a Bürker chamber. Results are always shown with respect to the corresponding sham condition (normalized to 1).

### Trans-Epithelial Electrical Resistance (TEER) Measurements

For TEER measurements, 5 × 10^5^ Caco-2 cells were seeded 7 days before irradiation in 6-well plate coculture inserts (PET, 2 × 10^6^ pores/cm^2^) (Greiner Bio-One). TEER measurements were performed with voltmeter/ohmmeter EVOM (World Precision Instruments). TEER measurements have been performed before the irradiation and then every hour, for the first 6 h post-irradiation, while subsequently a time gap of 3 h has been chosen up to 48 h post-irradiation both in presence/absence of coculture with PBMC.

### Cytokine Analysis

The amount of cytokine in the culture medium was analyzed using the Human Cytokine Array (RayBiotech), according to the manufacturer instruction. Then, 5 × 10^5^ cells were seeded 1 week before irradiation. Forty-eight hours after irradiation and both in presence/absence of PBMC in the basolateral compartment, supernatants were collected for cytokine quantification. Films were obtained after visualization with enhanced chemoluminescent kit (BioRad). Acquisition of films was performed with Gel Doc EZ Imager (BioRad). Identification of regions of interest and evaluation of fold changes (FC) were performed with Image Lab 4.0 software (BioRad). Three biological replicates were pulled together prior to the cytokine analysis.

### Western Blot Analysis of Claudin-1, Occludin, Afadin, ZO-1, ZO-2, NF-κB, and X-Linked Inhibitor of Apoptosis Protein (XIAP)

For sampling of cellular extract for western blot analysis, 5 × 10^5^ cells were seeded 1 week before irradiation. Forty-eight hours after irradiation and both in presence/absence of PBMC in the basolateral compartment, cells were lysed with Cell Lysis buffer (Cell Signaling Technology) following the manufacturer instruction and cellular extracts were stored at −20°C. Total protein quantification was performed with BCA method (Abcam) according to manufacturer instruction.

Proteins were mixed with Laemli Sample Buffer (BioRad) additionated with β-mercaptoethanol (BioRad) and heated at 95°C for 5 min, then centrifuged few seconds at 10,000 *g*. The same amount of proteins underwent electrophoresis in 4–20% precast gels (BioRad), and subsequently proteins were transferred on PVDF membranes (BioRad). After the blocking step with non-fat dry milk 5% in PBS 0.2% Tween-20, membranes were incubated overnight with primary antibodies: anti-claudin-1, anti-ZO-1, anti-ZO-2, anti-afadin (Cell Signaling Technology), anti-occludin (Millipore), anti-NF-κB (Epitomics), and anti-XIAP (Abcam). Samples were then incubated with anti-rabbit or anti-mouse HRP-conjugated secondary antibody (Amersham). Films were obtained after visualization with enhanced chemoluminescent kit (BioRad), and scanned with Gel Doc EZ Imager (BioRad). Finally, bands were quantified with Image Lab 4.0 software (BioRad).

### Statistical Analysis

For all the different experiments, each value represents the mean of at least three independent measurements ± SEM. To determine if the radiation dose and the coculture determine a statistically significant differential response, two-way ANOVA test with multiple comparisons for repeated measurements (with Bonferroni *post hoc* tests to compare replicate means) was performed. Where not otherwise stated, statistical significance (*p*) was calculated by two-tailed Student’s *t*-test.

## Results

### Effects of Irradiation on Caco-2 Proliferation

Cells were tested for proliferation and mortality after different doses of X-rays.

**Figure 1:**
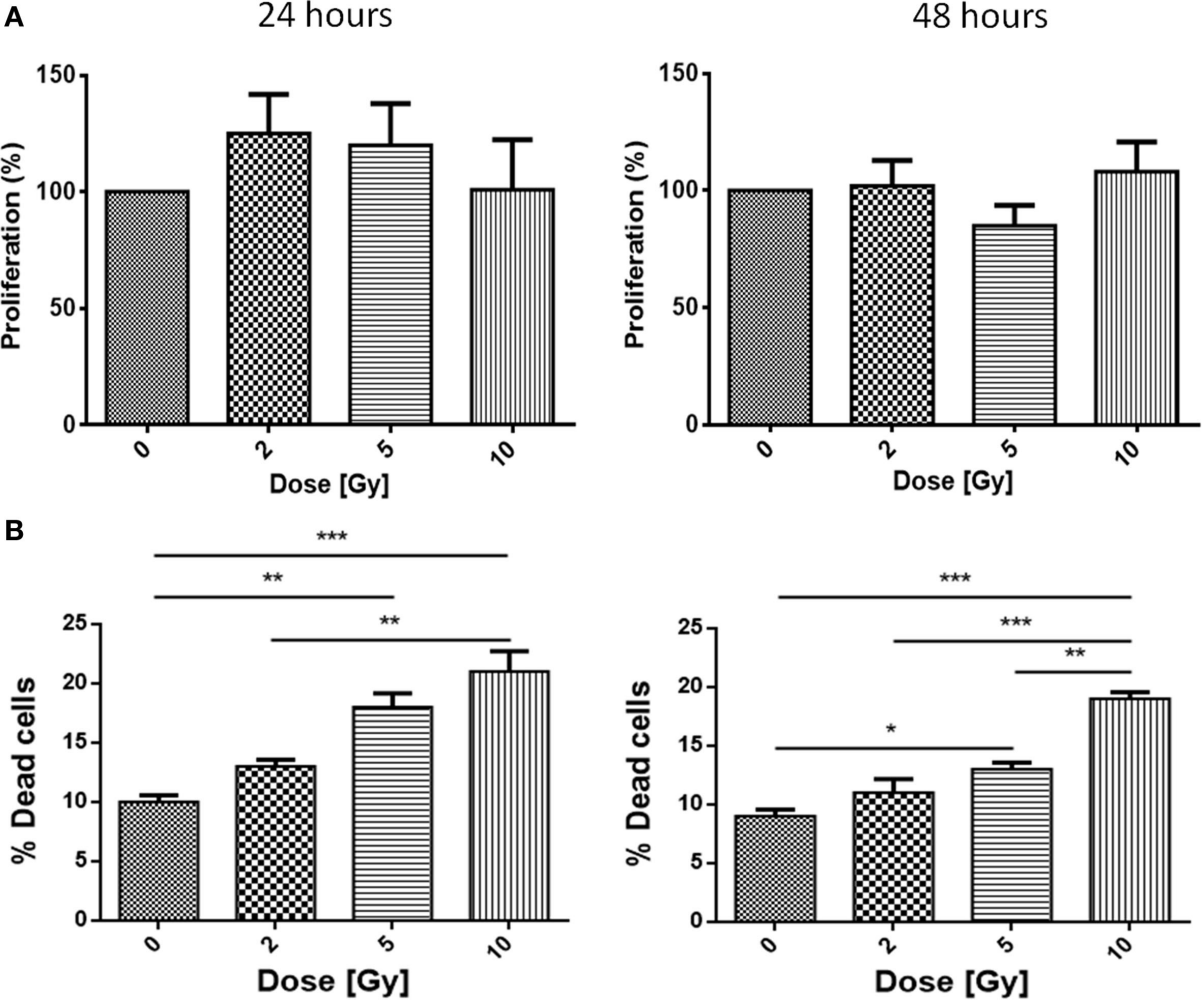
**Proliferation (A) and mortality (B) in Caco-2 cells exposed to 0, 2, 5, and 10 Gy of X-rays**. Each value is the mean of at least three independent experiments ± SEM.

Caco-2 cells were exposed to 0, 2, 5, and 10 Gy of X-rays. Having as reference the proliferation of the sham irradiated as 100%, 24 h post-irradiation a slight increase in proliferation is observed for all conditions, even if not statistically significant, with values reaching 125% for the 2 Gy and 120% for the 5 Gy. After 48 h, no differences persist for all the conditions with respect to the sham (Figure 1A).

Caco-2 cells were also tested for the mortality after irradiation. Cell mortality shows a dose-dependent trend; at 24 h, sham cells present a mortality level of 11%, while the 2, 5, and 10 Gy-irradiated cells show percentages of 13, 19, and 21%, respectively. Percentages after 48 h confirm the trend already observed for the 24 h samples: while the sham is characterized by a very low mortality (9%), mortality in irradiated samples is found to be 11% (2 Gy), 14% (5 Gy), and high statistical significance is associated to mortality after 10 Gy (20%) (Figure 1B).

### Evaluation of TEER Changes in Caco-2/PBMC Coculture after Radiation Exposure

Caco-2 cells were irradiated at confluence with doses of 0, 2, and 10 Gy. Half of the samples were cocultured with PBMC, and half were cultured alone on the same insert, but with no PBMC in the lower compartment. The TEER of the Caco-2 layer was monitored for up to 48 h, and an initial measurement was carried out right before the radiation exposure, to have a control for possible transient effect related to the irradiation protocol itself.

**Figure 2:**
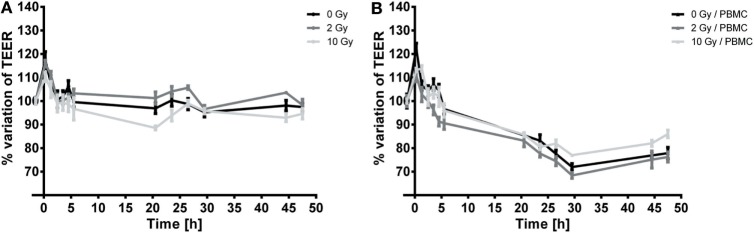
**Temporal dynamics of TEER in Caco-2 cells exposed to 0, 2, and 10 Gy of X-rays cultured alone (A) or cocultured with peripheral blood mononuclear cells (PBMC) (B)**. Each value is the mean of at least three independent experiments ± SEM.

In Figures 2A,B, we report the percentage variation of TEER after radiation exposure with respect to the pre-treatment conditions (measured values for pre-treatment TEER were found ranging between 300 and 350 Ω cm^2^), respectively for Caco-2 cells alone and in coculture with PBMC.

In both cases, a transient peak is observed, which can be ascribed to environmental/procedural stress related to the transportation to the irradiation facility. When such transient effect is over, the temporal dynamics of TEER can be considered as governed by the cell response to radiation.

When not cocultured with PBMC (Figure 2A), TEER values for the sham and 2 Gy conditions remain approximately constant as a function of time over the 48-h period under investigation. Conversely, cells irradiated with 10 Gy show a slight, but persistent, decrease in TEER starting from 3 h post-irradiation until 20 h post-irradiation, down to approximately the 90% of the initial TEER value; after such time point TEER grows again to values similar to the sham condition, and remains constant in the interval 24–48 h.

In presence of PBMC (Figure 2B), TEER values and evolution are significantly modified: after the transient increase, a decrease in TEER values starting from 3 h post-irradiation is observed for all conditions. TEER decreases roughly at a constant rate until approximately 30 h post-irradiation, and afterward settles at a constant value (about the 80% of the initial value) up to 48 h for the sham and 2 Gy conditions. In this case, cells irradiated with the 10 Gy seem to recover better than for the other conditions (as observable starting around 30 h post-irradiation), reaching at 48 h a TEER of about 86% of the pre-treatment value. The low effect of high doses radiation exposure in reducing proliferation of Caco-2 has already been described (20). Caco-2 cell are considered a radioresistant cell line, and radiation exposure could isolate clones with an enhanced resistance to X-rays. In support of this statement, Shin and colleagues demonstrated that, in the comparison between proliferation of different colorectal cancer cell lines (HCT-8, LoVo, WiDr, and Caco-2), Caco-2 cells proliferation was not affected after 10 Gy exposure. On the contrary, a 20% lower proliferation was found in HCT-8 and LoVo cells after 2 Gy exposure and after 6 Gy in WiDr cells (21).

### Analysis of Cytokine Release

**Figure 3:**
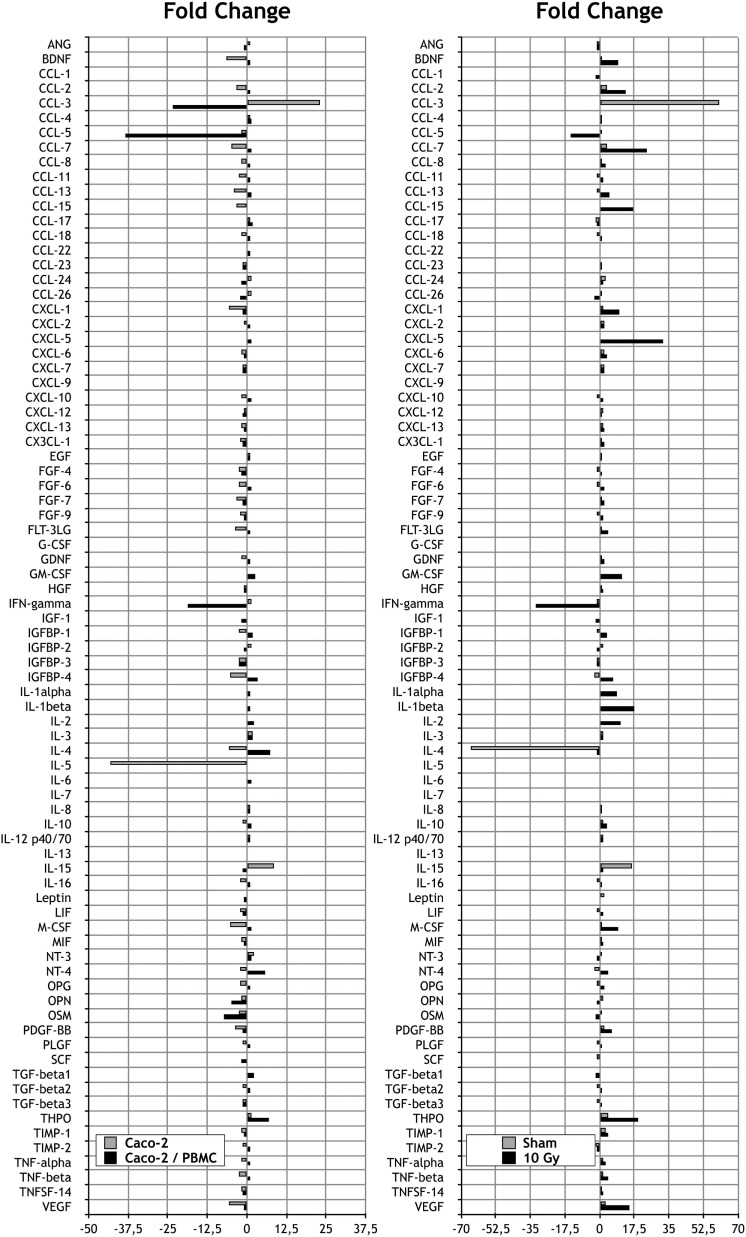
**Measurements of cytokine secretions in different experimental conditions**. Left: fold change (FC) analysis on Caco-2 exposed to 0 and 10 Gy: Caco-2 cultured alone (gray) vs Caco-2 cocultured with peripheral blood mononuclear cells (PBMC) (black). Each value is the ratio between the 10 Gy-irradiated samples and the corresponding sham. Right: FC analysis on Caco-2 cultured with or without PBMC: sham irradiated (gray) vs 10 Gy irradiated samples (black). Each value is the ratio between the cocultured vs not cocultured, at a fixed dose.

Focusing on the effects of radiation exposure with a fixed coculture setup, we first compared protein expression levels in culture media 72 h after 0 and 10 Gy-irradiated Caco-2 cultured in absence of PBMC. Such comparison shows that only three cytokines are released exclusively by cells not exposed to X-rays (Table 1), whereas the number of signaling proteins which can be found only when cells have been irradiated with 10 Gy increases to 10 cytokines (Table 1). We observed a reduction of the expression FC greater than approximately 5 in several cytokines families after radiation exposure: concerning the C–C and C–X–C chemokine, we found a reduction for CCL-7 (−4.9) and CXCL-1 (−5.8); we also observed a reduction of insulin-like growth factor-binding protein 4 (IGFBP-4, −5.2), interleukin-4 (IL-4, −5.4), interleukin-5 (IL-5, −43.2), macrophage colony-stimulating factor (M-CSF, −5.2), and vascular endothelial growth factor (VEGF, −5.5). Conversely, the results show an increase of the following cytokines: the C–C chemokine family protein CCL-3 (+23.1) and interleukin-15 (IL-15, +8.6) (Figure 3).

**Table 1:** **List of cytokines found in only one of the two irradiation conditions (sham vs 10 Gy) in the two coculture models [w/o vs with peripheral blood mononuclear cells (PBMC)]**.

W/o PBMC		With PBMC	
Sham	G-CSF	Sham	
	Leptin		
	SCF		
10 Gy	CCL-1	10 Gy	CCL-1
	CXCL-5		CCL-15
	CXCL-9		
	Granulocyte macrophage colony-stimulating factor		
	IGF-1		
	IL-13		
	Interleukin-1α		
	Interleukin-1β		
	Interleukin-2		
	TGF-β1		

The same analysis was repeated in Caco-2 cells exposed to radiation and then cocultured with PBMC. Differently from what observed in Caco-2 alone, we did not found cytokines expressed exclusively by sham-irradiated cells. On the other hand, we found the cytokines CCL-1 and CCL-15 released only in the coculture condition, with the 10 Gy-irradiated Caco-2 cells.

We observed a reduction of the protein expression FC greater than 5 in several cytokines families after radiation exposure: concerning the C–C and C–X–C chemokine we found a reduction for CCL-3 (−23.3) and CCL-5 (−38.3); we also identified the reduction of interferon-γ (IFN-γ, −18.8) and Oncostatin M (−7.2). Conversely, we observed an increase of the following cytokines: IL-4 (+7.5), neurotrophin-4 (+5.7), and thrombopoietin (THPO, +7) (Figure 3).

Trying to unravel the effects of coculturing Caco-2 with PBMC, we then focused on the comparison between cocultured and not cocultured cells for both the sham- and 10 Gy-irradiated conditions. Results for the sham-irradiated Caco-2 show that 3 cytokines are secreted only without coculture with PBMC, whereas the presence of PBMC induces the production and release in the culture medium of nine unique cytokines (Table 2). Following the exposure to 10 Gy of X-rays, three cytokines are found only in the Caco-2 w/o PBMC, while five are uniquely expressed in the coculture condition (Table 2).

**Table 2:** **List of cytokines found in only one of the two culturing setups [w/o vs with peripheral blood mononuclear cells (PBMC)] in the two irradiation conditions (sham vs 10 Gy)**.

Sham		10 Gy	
Caco-2 w/o PBMC	CCL-15	Caco-2 w/o PBMC	CXCL-9
	G-CSF		IL-5
	Interleukin-5 (IL-5)		IL-13
Caco-2 with PBMC	CCL-22	Caco-2 with PBMC	CCL-3
	CXCL-5		CCL-22
	Granulocyte macrophage colony-stimulating factor		IL-6
	IGF-1		Leptin
	Interleukin-1α		SCF
	Interleukin-1β		
	Interleukin-2		
	IL-6		
	TGF-β1		

Always, considering the comparison between Caco-2 cells cocultured or alone, we observed the increase in the expression levels, FC greater than approximately 5, in several cytokines families: concerning the C–C and C–X–C chemokine, we found an enhanced release of CCL-3 (+60.3) in the sham, while increases in CCL-2 (+13.2), CCL-7 (+23.8), CCL-15 (+16.7), CXCL-1 (+10), and CXCL-5 (+32.2) were observed in the 10 Gy irradiated. Considering other cytokine families, we detected a higher expression level of granulocyte macrophage colony-stimulating factor (+11), IGFBP-4 (+6.8), interleukin-1α (IL-1α, +9), interleukin-1β (IL-1β, +17.7), interleukin-2 (IL-2, +10.5), M-CSF (+9.1), THPO (+19.5), TNF-α (+3.3), and VEGF (+15.3) in irradiated cells and an enhanced production of IL-15 (+16.1) in sham irradiated.

On the contrary, Caco-2 cultured alone showed higher value of IL-4 (−65.2) when not irradiated, while higher values of CCL-5 (−14.6) and IFN-γ (−32) were found for the irradiated condition.

### Analysis of Proteic Levels of Claudin-1, Occludin, ZO-1, ZO-2, and Afadin in Caco-2

**Figure 4:**
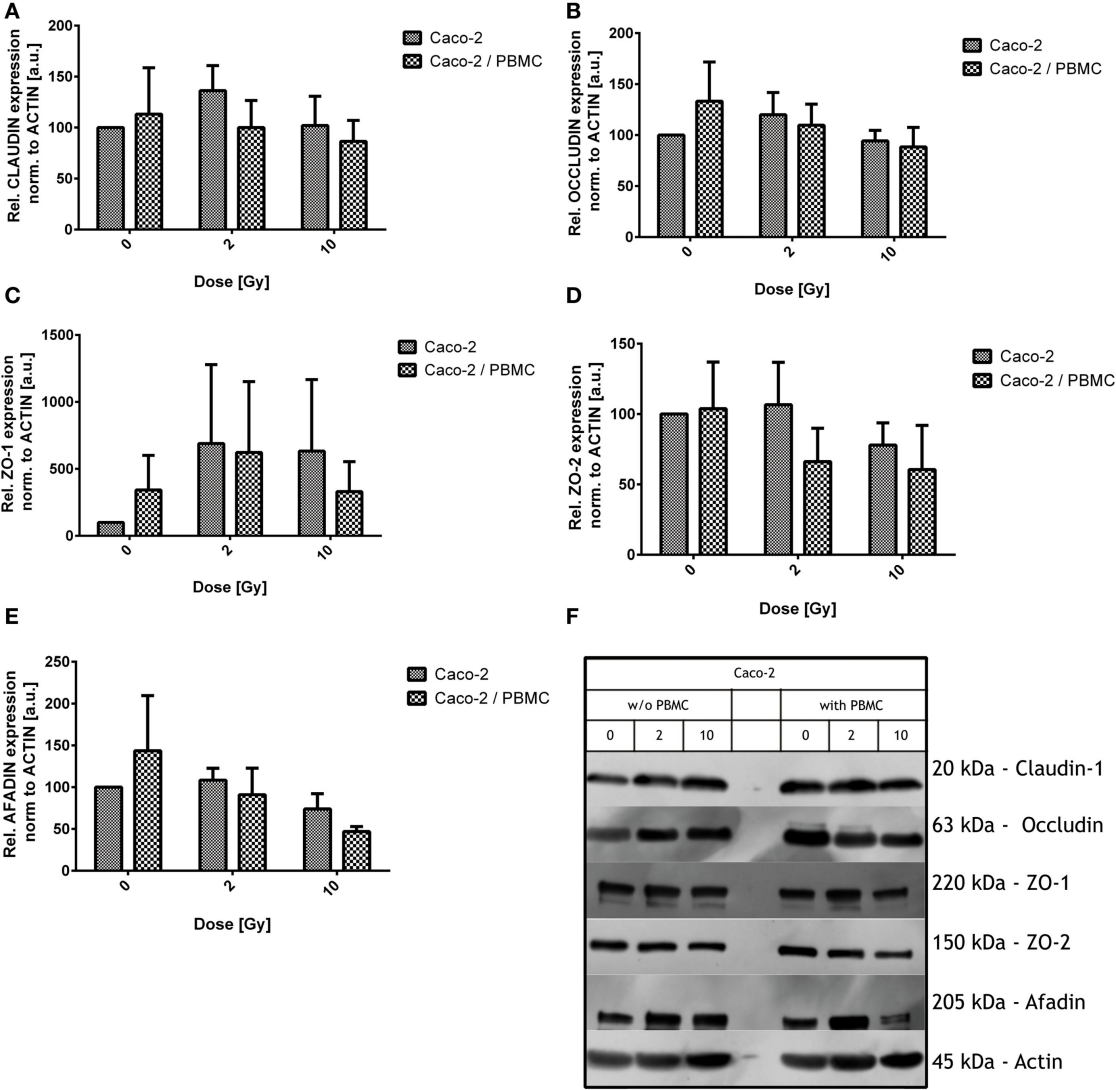
**Expression level of the tight junction proteins in Caco-2 cells for all irradiation conditions (0, 2, and 10 Gy) and with/without peripheral blood mononuclear cells (PBMC) in coculture**. Claudin-1 **(A)**, occludin **(B)**, ZO-1 **(C)**, ZO-2 **(D)**, and afadin **(E)**. Values are normalized on actin level. Each value is the mean of at least three independent experiments ± SEM. Representative films for each protein and conditions are shown in panel **(F)**.

We investigated the amount of proteins involved in tight junction complexes (claudin-1, occludin, ZO-1, ZO-2, afadin) in Caco-2 cells alone and in coculture with PBMC, 48 h after exposure for all irradiation conditions. All results are collected in Figure 4.

Claudin-1 expression was found quite stable among all the differently treated samples after irradiation, both in presence/absence of PBMC (Figure 4).

As observed for claudin-1, also occludin expression was not significantly modified by X-ray exposure and/or by PBMC coculture. Observed variations were found to be not statistically significant (Figure 4B).

Given the absence of modifications in directly related tight junction proteins, we moved to scaffold proteins. Expression levels of such proteins were modified by both radiation exposure and PBMC coculture (Figures 4C–E).

Large fluctuations are observed in ZO-1 protein levels; however, some considerations can be done: ZO-1 level is increased for cocultured Caco-2 with respect to Caco-2 alone in absence of radiation dose; exposure causes an increase of ZO-1 in both culture conditions, with similar expression levels at 2 Gy. Concerning not cocultured cells, no further change in ZO-1 level is observed when increasing the dose from 2 to 10 Gy. When cells are cocultured instead, the 10 Gy condition shows no difference with respect to the sham.

ZO-2 level seems to be reduced by radiation only for the 10 Gy condition in absence of PBMC, while, starting from 2 Gy, a decreased expression level is found in the coculture and remains constant also at 10 Gy. The difference in the culture condition only does not translate into a difference in ZO-2 expression.

Afadin is not affected after exposure to 2 Gy of X-ray. The 10 Gy-irradiation causes a reduction of afadin compared to the sham in absence of PBMC; the presence of PBMC enhances this effect, leading to a reduction of the 50% compared to what observed in the sham not cocultured cells (Figure 4).

### Analysis of Proteic Levels of XIAP and NF-κB in PBMC

Peripheral Blood Mononuclear Cells were cocultured for 48 h with Caco-2 cells irradiated with a dose of 0, 2, and 10 Gy, prior to their collection and cell lysis.

**Figure 5:**
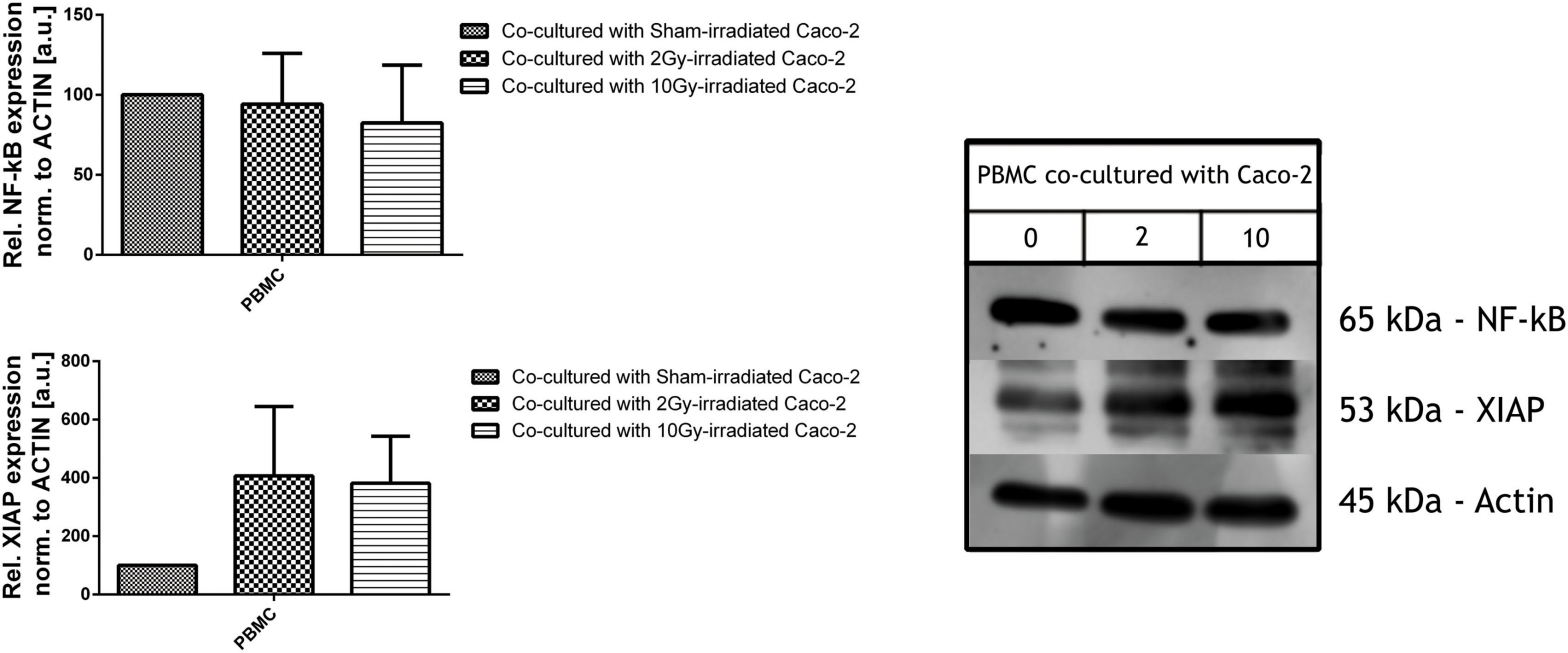
**Expression level of inflammatory and anti-apoptotic proteins NF-κB (upper panel) and X-linked inhibitor of apoptosis protein (XIAP) (lower panel) in peripheral blood mononuclear cells (PBMC) cocultured with not irradiated or irradiated (2, 10 Gy) Caco-2 cells**. Values are normalized on actin level. Each value is the mean of at least three independent experiments ± SEM.

Total amount of NF-κB was found not to be altered by the coculture with differently irradiated Caco-2 cells, while the expression levels of XIAP were upregulated in all PBMC cocultured with irradiated cells, both with 2 and 10 Gy: XIAP levels in such conditions were approximately four times higher than for the coculture with sham-irradiated cells, although large variations suggest that an higher number of samples is necessary to improve the statistics (Figure 5).

## Discussion

Colorectal cancer is one of the most important disease affecting industrialized society. Management of this type of tumor is routinely performed through surgery, chemotherapy or radiotherapy. Among these three methods, radiotherapy offers the opportunity to target the tumoral tissues limiting radiation exposure of healthy tissues.

In the last years, a plethora of data shed light on the so-called “bystander” effects, i.e., the radiation effects observed far from the irradiated target, and on the role played by the immune system in fighting against cancer concomitantly with radiotherapy, leading to radio-immunotherapy (2, 20–24).

Within this context, in this work we analyzed the effects of IR exposure with X-rays in colon adenocarcinoma Caco-2 cells, adopting a coculture experimental setup which allows to investigate the interplay between Caco-2 and PBMC.

First of all, we evaluated proliferation and mortality in Caco-2 cells after different doses of X-rays. Although proliferation (assessed with MTT, Figure 1A) did not decrease after radiation exposure, cell mortality (Trypan Blue, Figure 1B) was found to increase in a dose-dependent way up to 10 Gy of X-rays. Even though the increase was statistically significant, absolute values are very low. From the results of these assays, being MTT and Trypan Blue good complementary indicators of radiosensitivity (25), we evaluated the proliferation of viable cells normalizing the MTT results on the percentage of living cells obtained by Trypan Blue staining. Our calculations show an increased proliferation up to approximately 130% at 24 h after 5 Gy-irradiation. Considering all the irradiation doses and both time points, we can state that Caco-2 cells show a high radioresistance in this range of doses, with no severe effects on cellular proliferation or mortality.

The temporal dynamics of the trans-epithelial electrical resistance (TEER) of Caco-2 cell layer was then assessed (Figure 2). Being also present in sham-irradiated cells, the peak in TEER values observed within the first 2–3 h after irradiation can easily be ascribed to the stress induced by the transport to the irradiation facility. According to data available in the literature, we observed a decrease in TEER for the highest 10 Gy dose up to 24 h, while we did not observe changes in 2 Gy-irradiated cells with respect to the sham condition in the same time interval. After the 10 Gy-induced continuous decrease, a recovery process starts and such recovery appears to be completed within 48 h post-irradiation. TEER values for all irradiation conditions at 48 h are back to the pre-treatment level.

The presence of PBMC consistently changes the above described behavior. The same transient peak ascribable to environmental/procedural stress is observed and vanishes in few hours after irradiation. Our results demonstrate that a strong decrease in TEER values for all the irradiation conditions takes place over 30 h post-irradiation; after this time point, recovery starts. Interestingly, data show that the 10 Gy-irradiated cells recover to higher TEER values with respect to other conditions.

Comparing TEER values for Caco-2 with/without PBMC in coculture, we can conclude that the permeability of the Caco-2 layer seems to be strongly affected in presence of cells from the immune system. The mere presence of PBMC induces indeed a significant increase of permeability of the Caco-2 layer (decrease of TEER) over 48 h, which could be interpreted as due to the interplay between tumoral cells and the immune system. The situation is not changed if Caco-2 cells are exposed with 2 Gy X-rays, while a higher dose of 10 Gy seems to induce a shorter term (24 h) decrease in TEER in absence of PBMC, but a higher recovery to a less permeable layer at longer times (48 h) in presence of PBMC. A radiation effect appearing only at 10 Gy is consistent with the high radioresistance of Caco-2 derived from proliferation and mortality.

Expression of several cytokines from Caco-2 cells is differentially modulated depending both on the radiation exposure and the presence of PBMC (Figure 3). Concerning the family of interleukins, it is evident that the major effect observed is on IL-4, IL-5, and IL-15. Our results show that IR and coculture add their effects in the case of IL-4, while radiation only acts in reducing release of IL-5; in this case the presence of PBMC completely stops the release of this cytokine. In the case of IL-15, IR and the presence of PBMC taken alone increase the release, and all together the synergic effect is highlighted.

Taken together, these data highlight the modulation of IR and the cross talk between the two different components of the coculture. Considering the effects of measured interleukins, we can speculate an enhanced effect on proliferation of NK cells due the synergic effect on IL-15. In addition, the decrease of IL-4 could lead to the deregulation of the Th1 and Th2 subset of CD4^+^ T cells, with the reduction of Th1 cells, as highlighted in the work done by Pellegrini et al. (26). Of particular interest is the behavior of IGFBP-4; this cytokine is known to cause an increase in apoptosis and Bax protein expression, and a decrease in tumor cellular mitosis (27). While radiation and coculture taken alone reduce the production of IGFBP-4, the presence of PBMC with irradiated cell translates into an increased production of this protein: also radiation itself causes an enhanced release in the coculture setup. Of great interest is also the modulation of IFN-γ and TNF-α. IR and coculturing seem to have a synergic effect in decreasing the release of IFN-γ, and, on the other hand, in increasing TNF-α. The modifying action of IFN-γ on tight junctions is recognized, although a pleiotropic effect has been demonstrated for this cytokine. In studies about inflammation, direct treatment with IFN-γ acts increasing the paracellular permeability of endothelial and epithelial monolayers. However, in airway epithelial cells, IFN-γ exposure has anti-inflammatory properties and promotes epithelial barrier function (28, 29). For the interpretation of the enhanced TNF-α release, we recall that Ma and colleague showed that TNF-α exerts late effect on Caco-2 cell permeability, with an increased small-molecule flux within 24 h of treatment, and TEER alteration at 48 h post-treatment (30).

On the other hand, studies performed on normal colon cells showed a significantly higher amount of IFN-γ and TNF-α in inflamed-induced murine model causing, as a consequence, an enhanced proliferation in intestinal epithelial cells (31–33).

Data obtained through western blotting showed that the typical tight junction proteins claudin-1 and occludin are not affected by both radiation exposure and coculture. On the contrary, we observed changes in the scaffold proteins ZO-1, ZO-2, and afadin (Figure 4). ZO-1 is a fundamental scaffold protein which provides a binding site for a plethora of other proteins, i.e., ZO-2, ZO-3, occludin, and F-actin; its function is then mandatory to connect cellular cytoskeleton to the junctional complexes. Differently from Youakim and Ahdieh (34), who observed a mis-localization of ZO-2 after IFN-γ treatment, we observed a reduction in the levels of this protein. An effect on afadin was also found. Changes in the amount of ZO-2 and afadin after coculturing suggest the influence of coculture on the scaffold system, whose function is mandatory for the tight junction efficiency. The alteration observed in our setup could be explained by the changes in the observed values of the scaffold proteins. We can suppose such effects being mediated by the cross talk between Caco-2 cells and PBMC, in particular when Caco-2 cells are irradiated. Two of the major player of this cross talk can be easily identified in the INF-γ and TNF-α.

For what concerns the PBMC activation in response to Caco-2 cells coculture, we studied NF-κB and XIAP. NF-κB is a family of pleiotropic transcription factors which can be found in almost all cell types and it is involved in a myriad of cell functions, among which inflammation, immune response, and apoptosis. Exogenous signals triggering the cell surface receptors activate the canonical NF-κB signaling pathway, which first leads to the phosphorylation of the NF-κB-bound inhibitor (IkB), the nuclear translocation of the NF-κB dimers, mostly p65-containing heterodimers, and finally binding to the DNA to allow the regulation of the transcriptional activity of the cell (35, 36). But NF-κB activity influences also other cross-linked pathways, such as the p53 and apoptotic pathway, through the up-regulation of genes encoding inhibitory proteins, e.g., A20 and XIAP. In particular, XIAP is a member of the inhibitor of apoptosis family of proteins (IAP), which arrests apoptotic cell death binding to caspase 3, 7, and 9.

In our *in vitro* model, western blot assays did not reveal any increment in the constitutive levels of NF-κB of PBMC in irradiated cocultures, but the increase in the XIAP expression levels allows us to hypothesize its induction by MDM2 (37) and the activation of the cross-linked NF-κB pathway (38), with a subsequent inhibition of the extrinsic apoptotic pathway through the inhibition of the caspase 3 and caspase 7 (39) (Figure 5).

**Figure 6:**
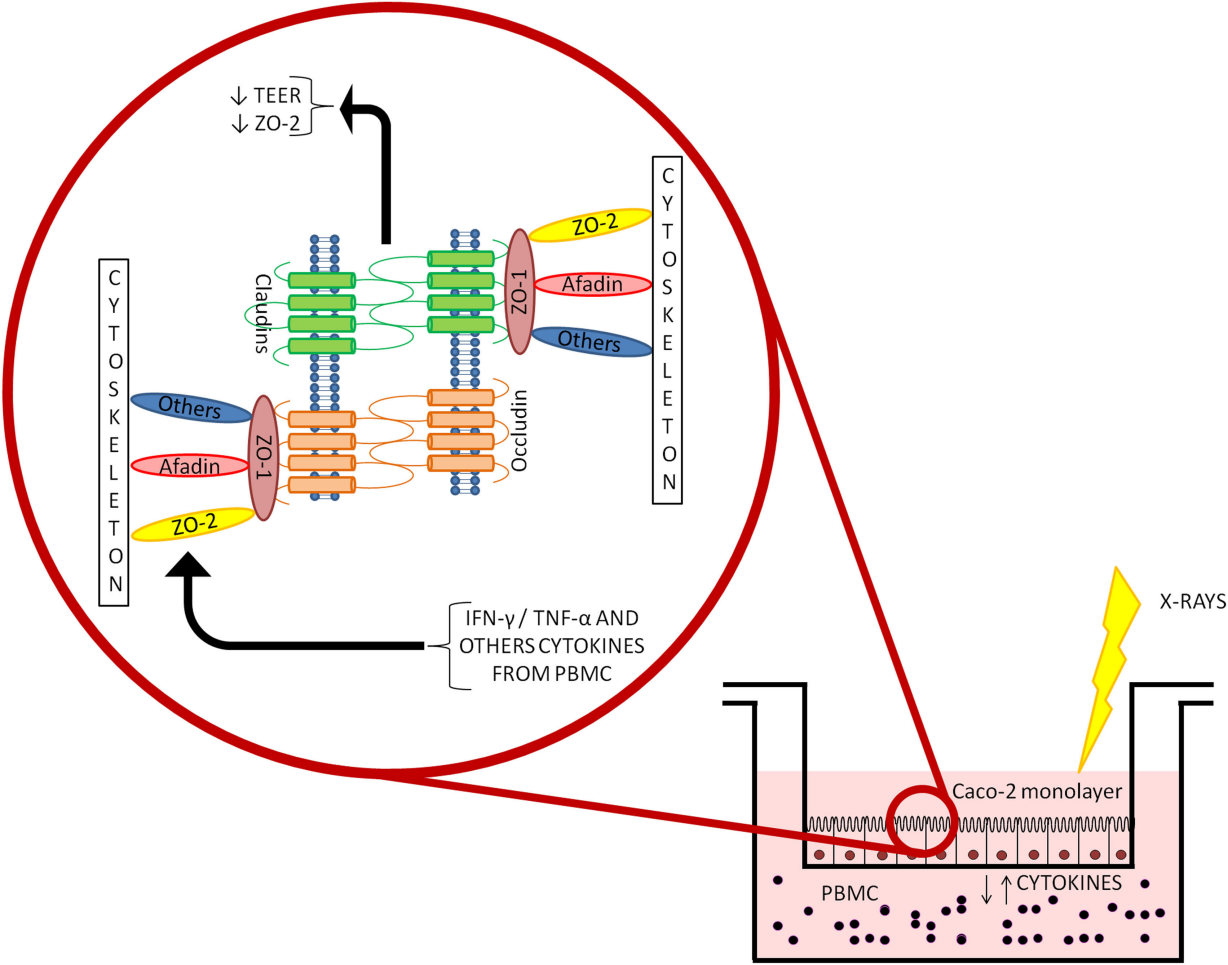
**Depiction of the coculture setup and focus on the mechanisms driving the altered permeability observed in Caco-2 cells cocultured with peripheral blood mononuclear cells (PBMC) after ionizing radiation exposure**.

In conclusion, our results show a weak effect of radiation on Caco-2 cells up to 10 Gy X-rays. The interplay with the immune system, addressed with a coculture setup with PBMC, induced increased permeability of the Caco-2 monolayer. Cocultured PBMC are activated, inhibiting the apoptotic pathway, therefore enhancing the release of cytokines in the culture medium. This could, in turn, affect tight junction scaffold proteins through IFN-γ- and TNF-α-mediated signaling (as illustrated in the simplified scheme of Figure 6).

## Author Contributions

JM, GBabini, GBaiocco, and AO: conceived the experiments. JM and GBabini: designed and performed all the experiments. JM, GBabini, GBaiocco, and SB: performed data analysis and data interpretation. JM, GBabini, GBaiocco, and SB: wrote and edited the manuscript. AO: critically read the manuscript.

## Conflict of Interest Statement

The authors declare that the research was conducted in the absence of any commercial or financial relationship that could be construed as a potential conflict of interest.
